# The Frontal Control of Stopping

**DOI:** 10.1093/cercor/bhv027

**Published:** 2015-03-09

**Authors:** Ashwani Jha, Parashkev Nachev, Gareth Barnes, Masud Husain, Peter Brown, Vladimir Litvak

**Affiliations:** 1 Sobell Department of Motor Neuroscience, UCL Institute of Neurology, London WC1N 3BG, UK; 2 Nuffield Department of Clinical Neurosciences, University of Oxford, Oxford OX3 9DU, UK; 3 Institute of Cognitive Neuroscience, UCL Institute of Neurology, London WC1N 3AR, UK; 4 Wellcome Trust Centre for Neuroimaging, UCL Institute of Neurology, London WC1N 3BG, UK

**Keywords:** conditional complexity, inferior frontal gyrus, magnetoencephalography, pre-supplementary motor area, stop-signal

## Abstract

Stopping is a critical aspect of brain function. Like other voluntary actions, it is defined by its context as much as by its execution. Its neural substrate must therefore reflect both. Here, we distinguish those elements of the underlying brain circuit that preferentially reflect contextual aspects of stopping from those related to its execution. Contextual complexity of stopping was modulated using a novel “Stop/Change-signal” task, which also allowed us to parameterize the duration of the stopping process. Human magnetoencephalographic activity and behavioral responses were simultaneously recorded. Whereas theta/alpha frequency activity in the right inferior frontal gyrus was most closely associated with the duration of the stopping process, earlier gamma frequency activity in the pre-supplementary motor area was unique in showing contextual modulation. These results differentiate the roles of 2 key frontal regions involved in stopping, a crucial aspect of behavioral control.

## Introduction

The ability to inhibit potential movements is considered to be a key aspect of higher-order brain function. While stopping may imply an absence of movement, it does not imply the absence of action. Like all actions, stopping is defined not only by the mechanics of its execution, but also by the conditions that attend it. Indeed, the importance of stopping usually lies in the *context*. Stopping may be more important if one hears a car horn while walking across a road than if one hears a coin drop from one's pocket. The neural network correlate of stopping must therefore invoke brain regions which are sensitive to the context, in addition to those responsible for executing the stop.

Stopping has been experimentally studied using the “Stop-signal” paradigm (Logan and Cowan 1984). Here, the subject is asked to quickly press a left or right button in response to a directional “Go” cue—the Go task. In a randomly intermixed number of trials, a second visual cue (the Stop-signal) is presented quickly after the first, instructing the subject to inhibit the planned response. During the task, both actions (going and stopping) are traditionally conceptualized as competing neural processes in a “horse-race”—the winner determining the participant's behavior on a trial-by-trial basis. The duration of the Go process is evident from the median reaction time (RT) of the Go task when no Stop-signal is presented. Although the duration of the Stop process cannot be measured directly, it can be inferred by experimentally varying the time with which the subject has to stop [the delay between the Go and Stop cues—termed the stimulus onset asynchrony (SOA)], and relating this to the success rate of stopping. This relationship between the timing of the stopping stimulus (SOA) and the success of the response is known as the inhibition function, and has a midpoint that represents the average duration of the Stop process—the Stop-signal reaction time (SSRT; Logan and Cowan 1984; Band et al. 2003).

Using variants of this paradigm, primate electrophysiological studies have demonstrated that signals in the premotor cortex (Mirabella et al. 2011) and supplementary motor area (SMA; Chen et al. 2010) have the potential to stop arm movements, and that signals in the frontal eye field (Hanes et al. 1998; Murthy et al. 2009) and superior colliculus (Paré and Hanes 2003) have the potential to stop saccades. However, human lesion studies have focused on two more anterior areas that have been shown to be essential for stopping: the pre-SMA (Isoda and Hikosaka 2007; Nachev et al. 2007) and the right inferior frontal gyrus (IFG; Aron et al. 2003; Aron and Poldrack 2006). The specialized functions of these two frontal areas during voluntary stopping remain unknown.

Specifically, no study has sought to dissociate their neural contributions in relation to a definitive feature of any voluntary action—the complexity of the conditions that attend it (Koechlin et al. 2003; Nachev et al. 2008). Just as more complex overt movements (e.g., “press the button now” versus the more complex “only press the button if you hear the bell”) will invoke different brain areas, so more complex covert stopping will do the same (e.g., “do not press the button now” versus the more complex “do not press the button only if you hear the bell”). Reasoning from the pre-SMA's well-established sensitivity to the conditional complexity of overt movements (Picard and Strick 2001; Nachev et al. 2008) and the right IFG's close involvement in modulating stopping time (Aron et al. 2004), here we hypothesize that the pre-SMA is preferentially sensitive to the *context* of stopping, whereas right IFG activity is related to the *mechanics* of its execution. We sought to dissociate the functions of these frontal regions by introducing novel contextual and execution-related parameters within the stop task.

## Materials and Methods

### Subjects and Paradigm

**Figure 1.**
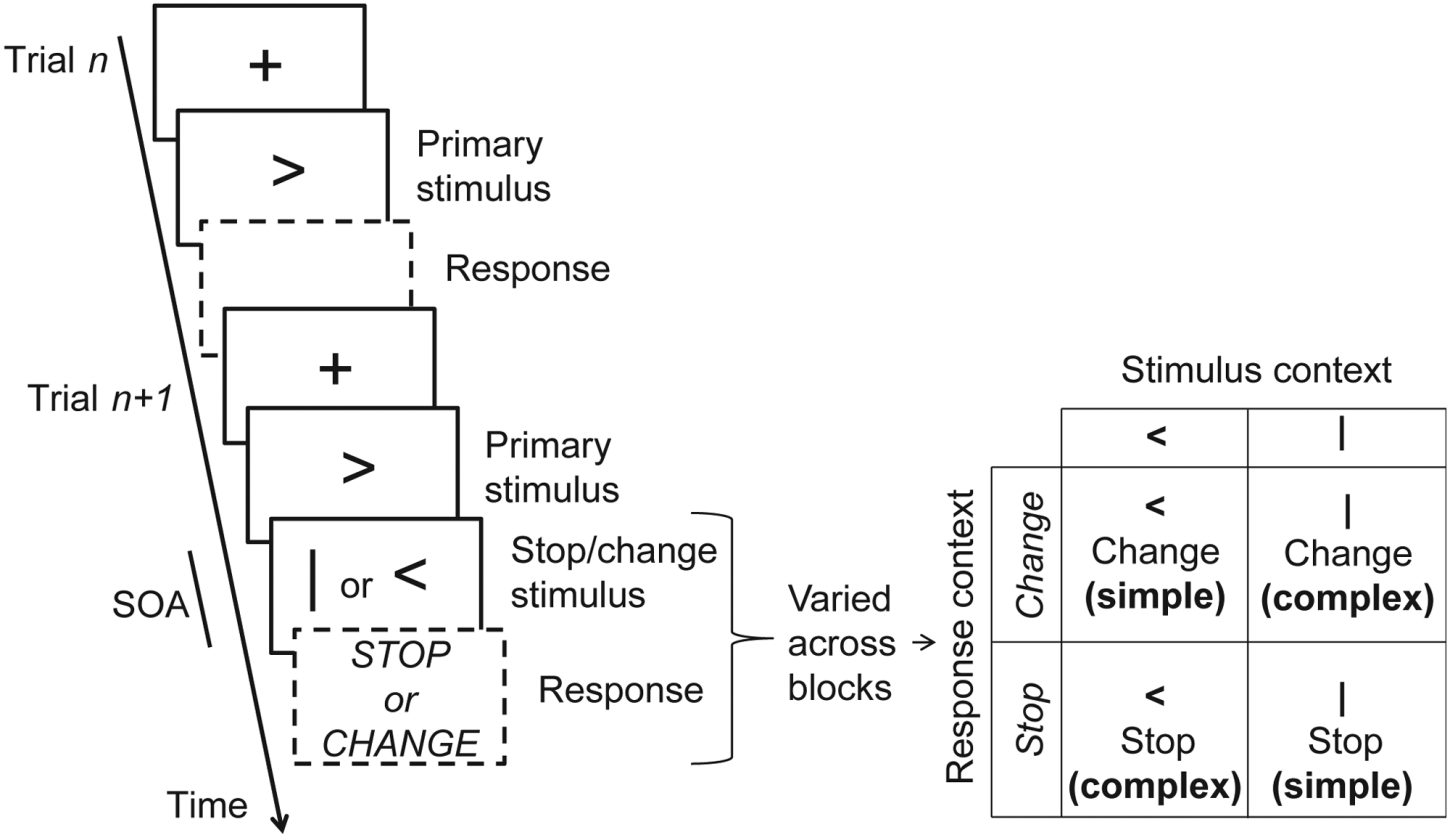
Paradigm details. We used 4 variants of the stop-signal paradigm arranged within a 2 × 2 factorial design. Each trial consisted of a left- or right- pointing Go arrow instructing the subject to press the corresponding button quickly (Go trials, e.g., trial *n* in figure). In 50% of trials, after a variable delay (the SOA), a further visual stimulus was presented. Depending on the variant of the paradigm (a single variant was used per task block), the subject was asked to either inhibit the planned response (Stop response context) or to inhibit the planned response, and additionally press the opposite button (Change response context), in response to the second visual stimulus. In addition to the response context, we manipulated the stop/change *stimulus* context by using either a vertical bar or a directional arrow (always opposite to the go-signal arrow) as the instruction to change or stop resulting in a factorial design (see table inset in right panel). The interaction between both manipulations isolates the effect of contextual complexity (see the main text and Table 1). The SOA was dynamically altered between trials to strive for a 50% correct response rate.

This study was approved by the Imperial College ethics committee. Nine healthy right-handed subjects [mean age 31 years (range 21–38 years); 5 females] were asked to perform 4 variants of the stop-signal task in a block design (see below and Fig. 1) while 275-channel magnetoencephalography (MEG) was acquired at 600 Hz by a CTF system (CTF/VSM MedTech, Vancouver, Canada). Two 4-min blocks of each variant were performed (the order counterbalanced across subjects) with a 1- to 2-min break in between blocks.

### Standard Stop-Signal Task

We will describe the standard stop-signal task first, before detailing the variants below. During each standard Stop-signal trial, the subject was presented with a fixation cross (lasting 1.3–1.5 s, the duration was drawn from a uniform distribution) which, after a 200-ms pause with a blank screen, was followed by a green left- or right- pointing arrow (the Go signal). The subject was asked to make a button press with the thumb of the corresponding hand as quickly as possible. In a randomly selected 50% of trials, a further red vertical bar was presented at a variable latency (SOA) after the Go signal. In response to this new signal, the subject was asked to inhibit the planned button press. The SOA was determined by a simple “staircase-type” adaptive algorithm (Levitt 1971), which randomly chose either of 2 values (set at 0 and 300 ms, respectively, at the outset) that were individually updated after each trial depending on its outcome: increasing by 50 ms after a successful Stop and decreasing by 50 ms after an unsuccessful Stop, asymptoting at zero. This adaptive algorithm targets the 50% performance level; 2 randomly sampled “threads” were used here [as in Nachev et al. (2005)] to reduce predictability from preceding trials.

### Approach to Manipulating Context of Stopping

To dissociate the effects of context, we used 4 variants of the Stop-signal paradigm arranged within a 2 × 2 factorial block design. They all had the same requirement for inhibition—the inhibition of the Go movement—but with 2 factors modulating context (Fig. 1). The notion of “context” in relation to a cued motor task is necessarily complex. Varying the sensory modality of the cue (e.g., visual vs. auditory), or the effector modality of the response (e.g., hand vs. eye), can all be explicitly viewed as manipulations of context. Such manipulations have been shown to affect both Go RT and SSRT (Logan and Irwin 2000; Morein-Zamir and Kingstone 2006).

Our focus here, however, is on a higher-order aspect of the context of action that is supervenient on the question of modality, either sensory or motor, for it applies regardless of it. Crucially, it is the aspect that most clearly differentiates actions within the voluntary domain—the complexity of the conditional relationship between the task stimuli and the task response (Koechlin et al. 2003; Nachev et al. 2008), what we shall call here *contextual complexity*—and is therefore of the greatest interest in relation to voluntary stopping. To isolate this crucial aspect of context, it is essential to keep the other aspects (potential confounds due to low-level contextual factors such as the sensory and effector modalities) the same or counterbalanced, as we seek to do here. It will be useful now to first describe the low-level experimental manipulations performed, before demonstrating how their interaction isolates an explicitly defined aspect of contextual complexity.

### Experimental Manipulation to Isolate Contextual Complexity

First, we manipulated the required *response*—instead of stopping the ongoing movement (e.g., “do not press the left button”), we required participants to change that plan to the alternative movement (“press right button instead of the left” in the above scenario). Note that in the latter (“Change-of-plan”) version of the task, visual cues remain the same as in the conventional Stop-signal paradigm, but the subject is required not only to inhibit the Go task but also to press the opposite button in response to the Stop-signal—now called a Change-signal in this context (Logan and Burkell 1986; Brown and Braver 2005; Nachev et al. 2007). It has previously been debated as to whether the Change-of-plan task is best conceptualized as a switch (where there are only two goals: “Go1” which does not complete and is replaced by “Go2”) or a Stop-signal variant (where there are three goals: “Go1” which is overridden by an active “Stop” and then “Go2” is initiated). However, recent detailed behavioral modeling studies have strongly supported the latter. Therefore, because both variants contain the same requirement to Stop, the stopping process can be compared across both the Stop-signal and Change-of-plan tasks (Camalier et al. 2007; Verbruggen et al. 2008; Verbruggen and Logan 2009).

Secondly, we manipulated the behavioral significance of the Stop/Change *stimulus*. We achieved this simply by using either a red vertical bar or a directional arrow (always opposite to the Go task arrow) as the instruction to change or stop (see Fig. 1 for table inset). The *interaction* between both manipulations isolates the effect of contextual complexity while controlling for any confounding differences between stopping and changing or the visual stimuli of the task. The critical manipulation of the context we are introducing here is contextual complexity. This was achieved by manipulating the *minimal number of informative elements that need to be interpreted* in order to successfully stop or change (Table 1).

**Table 1:** Complexity of rules when presented with a Stop/Change-signal in various task variants

Task variant	What to do when presented with particular combinations of Go cue and Stop/Change cue (highlighting the conditional complexity of task rules).
Stop-signal task (simple variant using “|”)	“>” and then “**|**” = stop, or… “<” and then “**|**” = stop *i.e., one cue “|” means stop regardless of preceding cue.*
Stop-signal task (complex variant using “<”)	“**>**” and then “**<**” = stop, or… “**<**” and then “**>**” = stop *i.e., the instruction to stop is presented only by combining* 2 *temporally closely related go-cues.*
Change-of-plan task (simple variant using “<”)	“>” and then “**<**” = press the left button “<” and then “**>**” = press the right button *i.e., only the later cue is needed to specify the action fully.*
Change-of-plan task (complex variant using “|”)	“**>**” and then “**|**” = press the left button “**<**” and then “**|**” = press the right button *i.e., the direction of change can only be inferred by combining the previous Go cue with the Change cue (it is opposite to the previous Change cue).*
	

Note: This table is discussed in the main text (see the “Experimental Manipulation to Isolate Contextual Complexity” section in Materials and Methods). In principle, interspersed Go trials, where only one Go cue is presented create the tendency to respond to isolated arrow cues (“<” or “>”) by pressing the corresponding button quickly. The 4 variants of the stop/change task manipulate the minimal number of informative elements that need to be combined to successfully Stop or Change. Some simpler variants require only one element (the Stop/Change-signal), whereas other more complex variants require 2 elements (the Go and Stop/change-signals) to be combined to infer the correct action. The minimal elements required in each case are highlighted in bold.

On any one trial, the participant is presented with a either single or double set of cues. They are asked to respond in one of only 3 ways: a left button press, a right button press, or a withheld button press (i.e., no motor response to the cues). On simple Go trials, a single left or right arrow Go signal instructs the participant to make a movement in the direction of the arrow. On double-cue trials, a left or right arrow Go signal is followed by a Stop- or Change cue, at a variable interval later, instructing the participant either not to perform the Go movement or to execute a movement in the opposite direction.

The single-cue trials, randomly interspersed among the others, serve to establish a proactive tendency to prepare a response to the first Go cue on the double-cue trials. Without them participants will simply tend to wait for the Stop/Change cue on double-cue trials, removing the preparation of a movement in response to the Go cue which the double-cue trials are designed to capture. One cannot have single-cue Stop or Change trials, for there would be nothing to stop or change.

Now, the critical manipulation of conditional complexity is the specification of the Stop or Change in the double-cue trials in terms of the minimal number of informative elements that need to be combined to successfully stop or change (Koechlin et al. 2003; Nachev et al. 2008).

Taking the Stop trials first, the simple variant of the task uses a Stop cue whose morphology unambiguously specifies that the participant has to stop. We do this by using a vertical bar symbol that does not appear in the context of the Go instructing symbols. The morphology of the cue (i.e., only one piece of information)—and no other aspect such as its timing—therefore unambiguously determines the action to be performed.

In the complex variant of the Stop task, the action is no longer unambiguously specified by the morphology of the cue but requires a further piece of information—its temporal order. We do this by using a similar “arrow” cue as is used for the Go cues, except that it is in the opposite direction to the Go cue presented earlier in the trial. Since the morphology is here shared with the Go cues, the temporal order—closely following a Go cue—is needed to specify the action. Two, rather than one, pieces of information are, therefore, required to specify the action.

Now consider how exactly the same manipulation of the cues alters the conditional complexity of a Change rather than a Stop version of the same task. In the Change-of-plan version of the Stop task, the participant has to change to the opposite response on double-cue trials rather than simply withholding the Go response. Now when the Change cue is an arrow, the direction of the Change is explicitly given: this is now the simple version of the task. When the Change cue is a vertical bar, however, the action is no longer unambiguously specified by the cue, for the direction is given as the opposite to that indicated in the preceding Go cue. This, therefore, becomes the complex variant, requiring 2 pieces of information (Table 1).

Note that since the cue asynchrony between Go and Change/Stop cues is typically only a few hundred milliseconds, this manipulation does not introduce a significant working memory load. How can we confirm that subjects are following our predicted rules? We would predict that more conditionally complex tasks are more difficult to perform and will have longer RTs. Therefore, if the participant follows the rules of the task as we expect, we will see a corresponding increase in the Go task RT in contextually complex conditions.

In short, we are thus able to introduce robust changes in conditional complexity with minimal manipulations of the underlying tasks in a way that reverses the direction of complexity across two paradigmatic tests of behavioral inhibition—Stop and Change—thereby eliminating potential confounding interactions. To achieve this, each subject was presented with a block of 155 trials of a single variant of the task before moving onto the next variant. The order of the 4 task variants was counterbalanced across the subjects.

### Behavioral Analysis

The aims of the behavioral analysis were 3-fold: to identify and exclude experimental runs where behavior was anomalous; to determine whether experimental modulations were evident in behavior (in particular whether contextual complexity resulted in an increased Go task RT); and to provide behavioral summary measures which could be used as predictor variables in the electrophysiological analysis. Stimuli and response timings were recorded and analyzed offline using custom Matlab scripts (The Mathworks, Inc., Natick, MA, USA), the Psignifit toolbox (Fründ et al. 2011), and IBM SPSS version 20.

Three key trial types were considered: Go-only trials (where the Stop/Change-signal is not presented), successful Stop/Change trials, and unsuccessful Stop/Change trials. Other trials, such as non-Stop/Change-signal trials where the left button was pressed in response to the right arrow were considered unclassified errors and discarded (Table 2). One subject was excluded because of excessive drowsiness (data not presented), and 2 conditions were discarded for another subject due to a software error (subject 1).

**Table 2:** Behavioral data for each subject

Subject	Condition	Presented trials	Unclassified trials	Classified trials	Change fraction	Fail fraction	Go RT	Fail RT	Success RT	Fail SOA	Success SOA	SSRTav	SSRTmcmc	Deviance
1	3	155	10	145	0.49	0.31	0.64	0.59	0.94	0.50	0.45	0.176	0.167	1.2
1	4	154	14	140	0.47	0.34	0.54	0.48	0.84	0.35	0.30	0.214	0.213	1.4
3	1	155	8	147	0.51	0.49	0.43	0.39		0.25	0.20	0.202	0.195	1.6
3	2	153	10	143	0.50	0.42	0.44	0.42		0.30	0.25	0.178	0.173	2.1
3	3	156	12	144	0.51	0.47	0.41	0.38	0.52	0.25	0.15	0.217	0.228	1.2
3	4	155	20	135	0.50	0.45	0.37	0.34	0.43	0.20	0.15	0.191	0.189	0.6
4	1	155	8	147	0.51	0.35	0.71	0.66		0.55	0.45	0.224	0.211	3.2
4	2	122	8	114	0.48	0.29	0.73	0.70		0.58	0.50	0.229	0.216	3.1
4	3	155	14	141	0.48	0.33	0.71	0.60	1.01	0.48	0.45	0.246	0.231	2.7
4	4	155	12	143	0.46	0.33	0.63	0.54	0.94	0.47	0.40	0.208	0.199	4.8
**5**	**1**	153	4	149	0.49	0.33	0.57	0.54		0.42	0.40	0.157	0.147	**7.9**
**5**	**2**	155	4	151	0.50	**0.20**	0.72	0.71		0.60	0.55			
5	3	155	12	143	0.48	0.35	0.78	0.68	1.09	0.60	0.50	0.245	0.245	2.2
**5**	**4**	155	36	119	0.42	**0.27**	0.63	0.50	0.79	0.40	0.35			
6	1	154	6	148	0.51	0.40	0.53	0.48		0.35	0.30	0.216	0.198	6.9
6	2	151	4	147	0.48	0.39	0.65	0.61		0.50	0.45	0.199	0.197	1.7
**6**	**3**	151	18	133	0.48	**0.23**	0.86	0.71	1.10	0.70	0.60			
6	4	155	12	143	0.50	0.37	0.76	0.69	0.95	0.60	0.50	0.233	0.209	1.4
7	1	156	10	146	0.49	0.42	0.54	0.48		0.40	0.35	0.178	0.169	7.6
7	2	154	6	148	0.49	0.40	0.50	0.47		0.35	0.30	0.161	0.168	5.6
7	3	155	16	139	0.49	0.43	0.55	0.50	0.72	0.35	0.30	0.221	0.213	2
7	4	155	14	141	0.49	0.44	0.61	0.56	0.85	0.45	0.40	0.191	0.194	0.8
8	1	155	7	148	0.49	0.42	0.43	0.42		0.25	0.25	0.170	0.165	1.3
8	2	155	8	147	0.50	0.38	0.54	0.50		0.30	0.30	0.256	0.243	1.3
**8**	**3**	154	31	123	0.44	**0.24**	0.74	0.56	0.86	0.50	0.45			
**8**	**4**	156	38	118	0.41	**0.24**	0.58	0.48	0.75	**0.32**	**0.33**			
9	1	154	10	144	0.48	0.44	0.57	0.53		0.30	0.25	0.306	0.300	4.3
9	2	154	8	146	0.50	0.36	0.56	0.47		0.35	0.30	0.216	0.189	2.5
9	3	154	25	129	0.50	0.35	0.60	0.52	0.67	0.35	0.30	0.251	0.222	2.2
9	4	156	37	119	0.47	0.34	0.53	0.51	0.61	0.35	0.30	0.178	0.163	2.9
Mean		**153**	**12**	**141**	**0.49**	**0.39**	**0.57**	**0.52**	**0.80**	**0.39**	**0.34**	**0.213**	**0.204**	**2.7**

Note: Data from subject 2 as well as conditions 1 and 2 from subject 1 were discarded due to drowsiness and a software error, respectively. Otherwise, each subject performed 4 variants of the task defined by the stop/change stimuli (| or >) and the response required (stop or change). The conditions were: (1) Stop |, (2) Stop <, (3) Change |, and (4) Change <. The total number of presented trials per condition is shown with the number of classified trials taken onto further analysis. Unclassified trials include trials where the subject lapsed or made unclassified errors (e.g., pressed 2 buttons). The change fraction is the fraction of change trials/total classified trials. The fail fraction is the fraction of unsuccessful trials/all change trials. The median RTs of Go task only trials (Go RT), unsuccessful Stop/Change trials (Fail RT), and successful Stop/Change trials (Succ RT) are presented. Successful Stop trials do not have a RT. Median SOA for successful and unsuccessful Stop/Change trials is presented. The Stop-signal RT was estimated using an average of two standard techniques (SSRTav) and also by deriving the midpoint of the inhibition function using a Bayesian MCMC procedure (SSRTmcmc). The goodness-of-fit of the MCMC model is quantified by Deviance which is a generalization of the sum-of-squares metric. Some subjects and conditions were rejected from further analysis (highlighted by bold) due to low fail fractions (<0.28), behavioral measures of SOA not in keeping with the horse-race model (if successful SOA > unsuccessful SOA), and poorly fitting psychometric functions (deviance >7.5). If a session was discarded on either of the first two grounds, then we did not proceed to derive a psychometric function. The mean of the data, after having removed the rejected sessions, is given in the bottom row.

To ensure that the task was performed in accordance with the assumptions of the horse-race model, we analyzed median RTs per subject per session. The horse-race model predicts that unsuccessful Stop/Change trials are faster than average and therefore unsuccessful Stop/Change RT should be faster than Go RT. Similarly, unsuccessful Stop/Change trials have higher SOAs on average than successful trials and this should be reflected in their relative median SOAs.

We performed two further analyses to check that remaining subjects had engaged in the task appropriately. First, we calculated the proportion of unsuccessful Stop/Change trials (fail fraction) per condition. Subjects performing the task correctly should have a fail fraction approaching 0.5 (50% of presented Stop/Change trials are successfully stopped or changed). Secondly, we modeled the inhibition function of each data run to make sure that it did not deviate excessively from the predicted form—any such deviation would suggest that the task was not performed correctly. The inhibition function models the proportion of correct responses, that is, successful Stops or Changes, as a function of the SOA, or the SOA corrected for RT (Go task RT—SOA; Logan and Cowan 1984; Band et al. 2003). We began by visually inspecting the SOA staircases to ensure that both SOA staircases converged (see Supplementary Fig. 1, top panel). Subjects tended to *wait* for the Stop/Change-signal, presumably to increase their success rate. This causes the Go task RT to drift upwards. Previous studies have suggested that the medial frontal cortex is sensitive to RT changes (Grinband et al. 2011; Yeung et al. 2011), therefore to isolate complexity effects from confounding RT drifts we used a method that considered the shape of the inhibition function and was comparable to (Nelson et al. 2010), to correct for RT drift. We compared the SSRT obtained from this method with two standard techniques (the average difference and integration methods) to ensure that our inhibition function-based analysis could reproduce the SSRTs captured by the former (see Supplementary Material).

To look for behavioral evidence of experimental modulations, median RT was estimated after the first and last 15 trials of each subject session were removed (to partially counteract RT drift). We were able to fully remove the effects of RT drift when only analyzing post Stop/Change trials by calculating a corrected post Stop-signal RT (current trial Go-only trial RT − previous Go-only trial RT). To increase trial numbers, we included trials where a Go-only trial was preceded by several Stop/Change trials, as long as the outcome for all Stop/Change trials was the same [similar to Bissett and Logan (2011)]. All behavioral measures including SSRT, RT, and adjusted RT data were subjected to mixed hierarchical general linear models (GLMs) with fixed-factors as described in the Results section, and subject as a random factor to look for significant behavioral changes.

### Magnetoencephalographic Data Preprocessing

**Figure 2.**
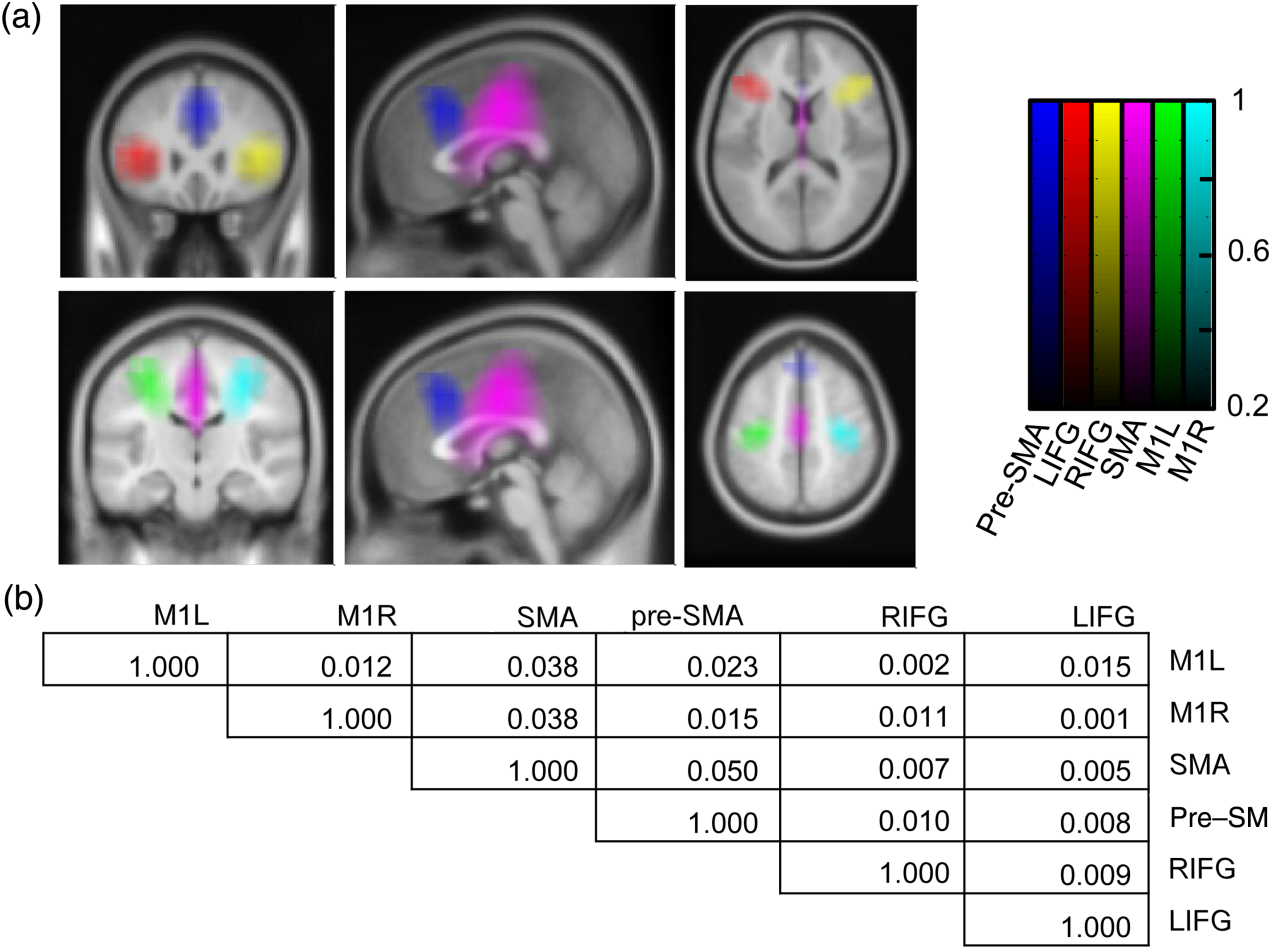
Regions of interest. (*a*) Beamformer filters for each location of interest were correlated with filters for the rest of the brain. These correlation images were averaged across subjects and then thresholded at *r*^2^ = 0.2. Each resulting image represents a maximal region of interest. Locations are the left (red) and right (yellow) inferior frontal gyrus, the pre-SMA (blue), the SMA (purple), and the left (green) and right (cyan) primary motor cortices. The color scale represents *r*^2^. (*b*) Individual correlations of filters between point source locations are presented and are very low. We have therefore been able to adequately separate the electrophysiological signal from the neighboring regions using the beamformer technique. Values represent *r*^2^. Locations are left (M1l) and right (M1r) primary motor cortex, SMA, pre-SMA, and right (rIFG) and left (lIFG) inferior frontal gyri.

MEG data were analyzed using SPM8 (Litvak et al. 2011) and Fieldtrip (Oostenveld et al. 2011) toolboxes. The data were down-sampled to 300 Hz, high-pass filtered above 0.1 Hz, and the line noise artifacts at 50 and 100 Hz were removed using notch filters (fifth-order zero-phase Butterworth filters). We then extracted time-series data from cortical regions of interest using a linearly constrained minimum variance (LCMV) beamformer (Van Veen et al. 1997). The MNI co-ordinates (*x*,*y*,*z*) of locations of interest were taken from the literature and included the pre-SMA [2,30,48 taken from Nachev et al. (2007)], the right and left IFG .[±42,26,14 adapted from Aron et al. (2007)], the SMA [−2, −10,59 adapted from Mayka et al. (2006)], and both the primary motor cortices [±37,−25,62 adapted from Mayka et al. (2006)]. Locations obtained from Mayka et al. (2006) were converted from Talairach to the MNI space using a transform devised by Mathew Brett (http://imaging.mrc-cbu.cam.ac.uk/imaging/MniTalairach). The beamforming method involves linearly projecting the MEG sensor data using a spatial filter computed from the lead field of the source of interest and the data covariance (Van Veen et al. 1997). The spatial filter is designed to extract activity from the region of interest, while suppressing activity from other sources. Lead fields were computed using a single-shell head model (Nolte et al. 2004) based on an inner skull mesh derived from a canonical *T*_1_ MRI. Data covariance matrices were computed using all the data from a recording block, for each block separately, and regularized by adding an identity matrix multiplied by a coefficient equal to 0.01% of the mean of the diagonal covariance matrix elements. The orientation of each source was specified to be in the direction of maximum power (Sekihara and Nagarajan 2004). To determine whether data from these sources were adequately separated, we correlated the beamformer filters (weights applied to the sensor data) of each source with all other sources per data run. This gave an average Pearson's *r* square value per subject describing the amount of variance at one location that could be described by the signal at another location due simply to the beamformer weights (i.e., non-physiological factors). The maximal mean percentage variance of variance explained was 5% between SMA and pre-SMA (Fig. 2*b*), confirming that the source reconstruction allowed us to discriminate between sources of interest.

Time-series data were then standardized by subtracting the mean and dividing by the standard deviation. To make standardization robust to possible artifacts, medians of the raw and squared signals were computed for non-overlapping 10 s segments and averaged yielding first- and second-moment estimates. Time-frequency representation of the data was generated using multitaper spectral analysis (Thomson 1982), and applied over whole blocks in time windows of 0.4 s shifted in steps of 0.05 s over a frequency range of 2.5–90 Hz in steps of 2.5 Hz. The frequency resolution was set to the inverse of the time window (2.5 Hz) for up to 25 Hz, then 0.1 times the frequency for 25–50 Hz, and then to a constant of 5 Hz. The power was transformed with the square root transform to obtain root mean square (RMS) amplitude, which better conforms to the linearity assumption of the convolution method (Litvak et al. 2012). RMS data were analyzed hierarchically: summary measures of induced responses were obtained with a first-level convolution model, then transformed into time-frequency images, and finally subjected to a standard GLM at the second level.

### The Convolution Model for Magnetoencephalographic Data

To characterize and disambiguate induced responses to the events of interest, regressors were generated for each event type, and assembled as predictors of continuous frequency-specific amplitude in a GLM. Each event was modeled as a delta function (an impulse) and then convolved with a set of Fourier basis functions spanning −0.5 to +1.5 s relative to each event (the peristimulus time window). GLM coefficients were estimated using ordinary least squares treating the different frequencies separately in a mass-univariate fashion. The induced response for a particular event type was reconstructed by multiplying the basis functions with a matrix of parameter estimates corresponding to the event type in question (Litvak et al. 2012). In the simplest case, with non-overlapping events, this would be equivalent to averaging time-frequency images centered on an event of interest. However, because our data contain multiple temporally overlapping responses, the convolution model was superior to event-locked routine averaging. Because, multiple predictors (different events) are included *in the same* GLM, the induced responses to different event types can be disambiguated from each other, if they do not always occur together. For example, the induced response to a Stop-signal during an unsuccessful Stop-signal trial can be disambiguated from the associated button press, allowing for a direct comparison of successful and unsuccessful responses to a Stop-signal. Individual regressors were specified for the fixation cross, the Go task stimulus, the button press responses (separately for left and right responses), and the Stop/Change-signal (separately for successful and unsuccessful Change conditions). Previous studies have suggested that the medial frontal cortex is sensitive to RT changes (Grinband et al. 2011; Yeung et al. 2011); therefore, we sought to include this confound in our model by using the predicted Go spline as a parametric modulator of the Go signal-induced response. We included the following regressors in our model: the mean Go event, a Go RT drift covariate, and separate regressors for all combinations of the current trial (left or right cue) and previous trial type (Go-only trial, successful Stop/Change trial, and unsuccessful Stop/Change trial). The resulting induced responses are independent of RT drift. Separate models were used to estimate induced responses during different Stop-signal task variants. The resulting time-frequency images from different tasks were later combined in a single second-level model in order to perform a statistical inference of the effects of interest (see below). The data and the design were filtered below 0.25 Hz.

### Analysis of Time-Frequency Images

**Figure 3.**
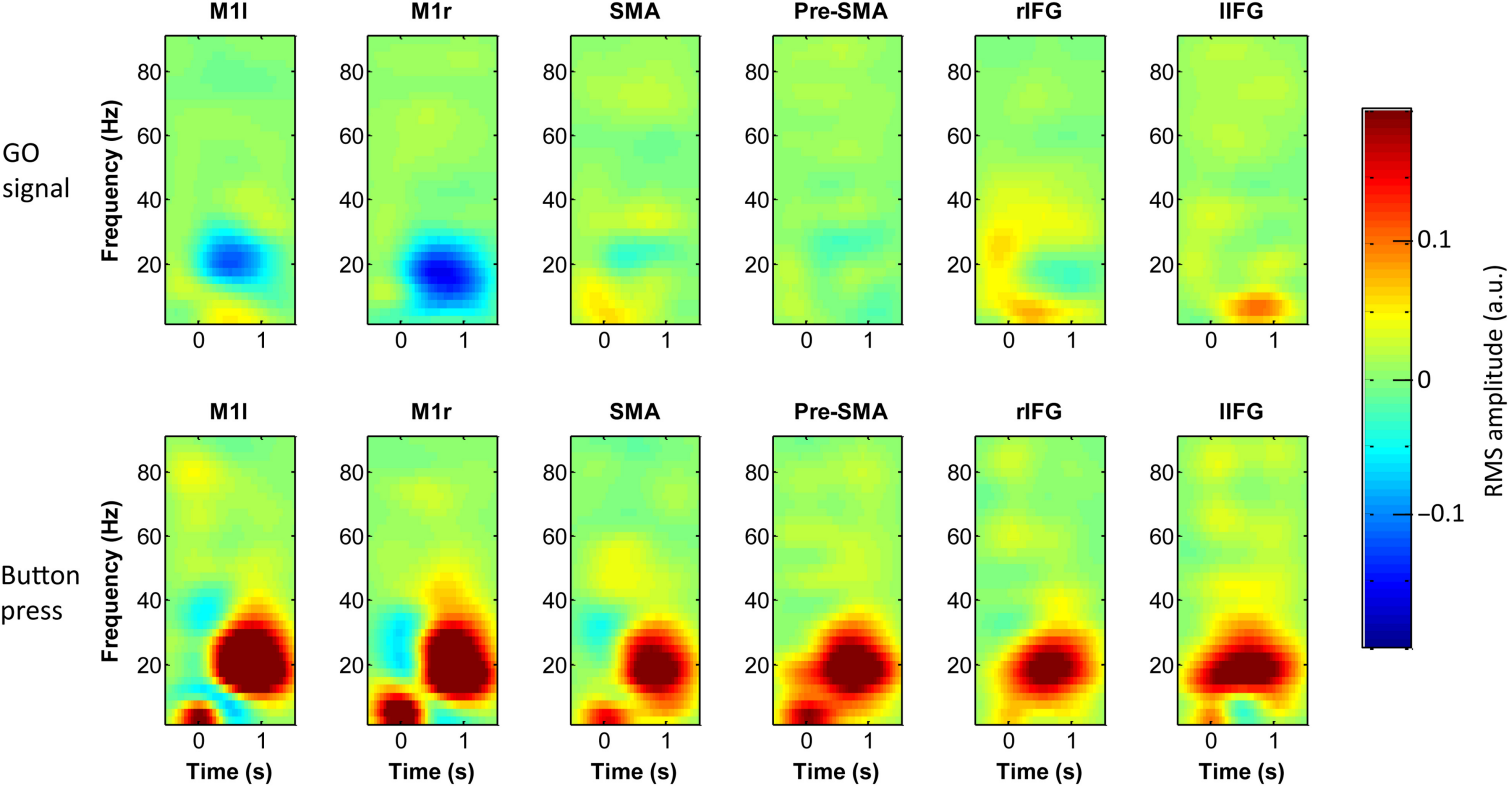
Estimated event-related activity from the convolution models of cortical activity. Each image is the induced response to the Go signal (top row, Go event is at time = 0), and to the button press (bottom row, button press is time = 0) at different cortical locations. Locations are left (M1l) and right (M1r) primary motor cortex, SMA, pre-SMA, and right (rIFG) and left (lIFG) inferior frontal gyri. Note that the button press causes a global induced response involving all areas tested. The color scale represents the RMS amplitude in arbitrary units.

After eliminating RT confounds, we generated time-frequency images for each event type and entered these into within-subject ANOVAs for each cortical source. Examples of mean induced responses to the primary Go signal and to a button press are shown in Figure 3. We studied responses to two event types statistically: the Stop/Change-signal—with success (successful or unsuccessful), Stop/Change stimulus (< or |) and response (Stop or Change) as factors, and the Go signal—with the current trial (left or right cue) and previous trial type (Go-only trial, successful Stop/Change trial, and unsuccessful Stop/Change trial) as factors. Two-tailed *t*-tests were performed for each main effect and interaction, and were thresholded at *P* = 0.05 FWE (peak-level), taking error non-sphericity into account using standard procedures (Litvak et al. 2011).

## Results

### Behavioral Data Meet Assumptions of the Horse-Race Model

Trial numbers and RT data are presented for each subject/session in Table 2. The mean RT for the Go task was 0.57 s (SD 0.11 s). To determine whether subjects followed the task appropriately we used 3 criteria. First, if the assumptions of the horse-race model are applicable to our task, then unsuccessful Stop/Change trials should reflect a faster than average subset of Go trials and indeed unsuccessful Stop/Change RT was less than Go RT for all subjects and sessions (Table 2). Additionally, unsuccessful Stop/Change trials should have a higher than average SOA and this is the case with all sessions except subject 8 session 4, which was, therefore, excluded from further analysis.

Secondly, subjects were instructed to perform the Go task as fast as possible and not “wait” for the Stop/Change-signal. Because of the SOA staircase procedure, an “ideal” subject would successfully stop or change only 50% of the time. In spite of this, overall most subjects still usually have a tendency to wait, increasing their proportion of successful trials. However, for some subjects this is either excessive, or they had a tendency to double-press/respond inappropriately to difficult Stop/Change trials (make unclassified errors). This manifests as a low fail fraction (the fraction of presented Stop/Change trials that are unsuccessful), and therefore we excluded subjects with a fail fraction arbitrarily below 0.28 (i.e., they were unsuccessful in only 28% of the presented Stop/Change trials) from further analysis. Mean fail fraction of the remaining subjects was 0.39.

Finally, we estimated SSRTav using the mean of 2 traditional methods—the average difference method and the integration method (see Materials and Methods and also Supplementary Methods). SSRTs were comparable across subjects and with the previous literature (Table 2). Using a Bayesian Markov Chain Monte Carlo (MCMC) procedure centered on SSRTav and correcting for an RT drift (Kuss et al. 2005; Fründ et al. 2011), we estimated the midpoint of the inhibition function (representing the difference between the SSRT calculated by the inhibition function and the SSRTav) for each subject and condition. The data from one subject/session fitted poorly to a sigmoid inhibition function (deviance greater than 7.5, see Supplementary Fig. 1, bottom right panel), suggesting that behavior during this session did not fit the horse-race model and was therefore excluded from further analysis. Using all these approaches, 5 of 30 sessions were excluded in total, leaving 25 remaining on which all further analyses were performed. Mean SSRTav was 0.213 s (SD 0.034 s), SSRT derived from the MCMC procedure was 0.204 s (SD 0.032 s), and the mean difference between the two estimates was 0.009 s (SD 0.010 s). We concluded that the traditional methods of calculating SSRT (SSRTav) and the MCMC procedure (designed to remove the RT drift) gave similar values of SSRT.

### Contextual Complexity Affects Behavior and Pre-SMA Gamma Activity

**Figure 4.**
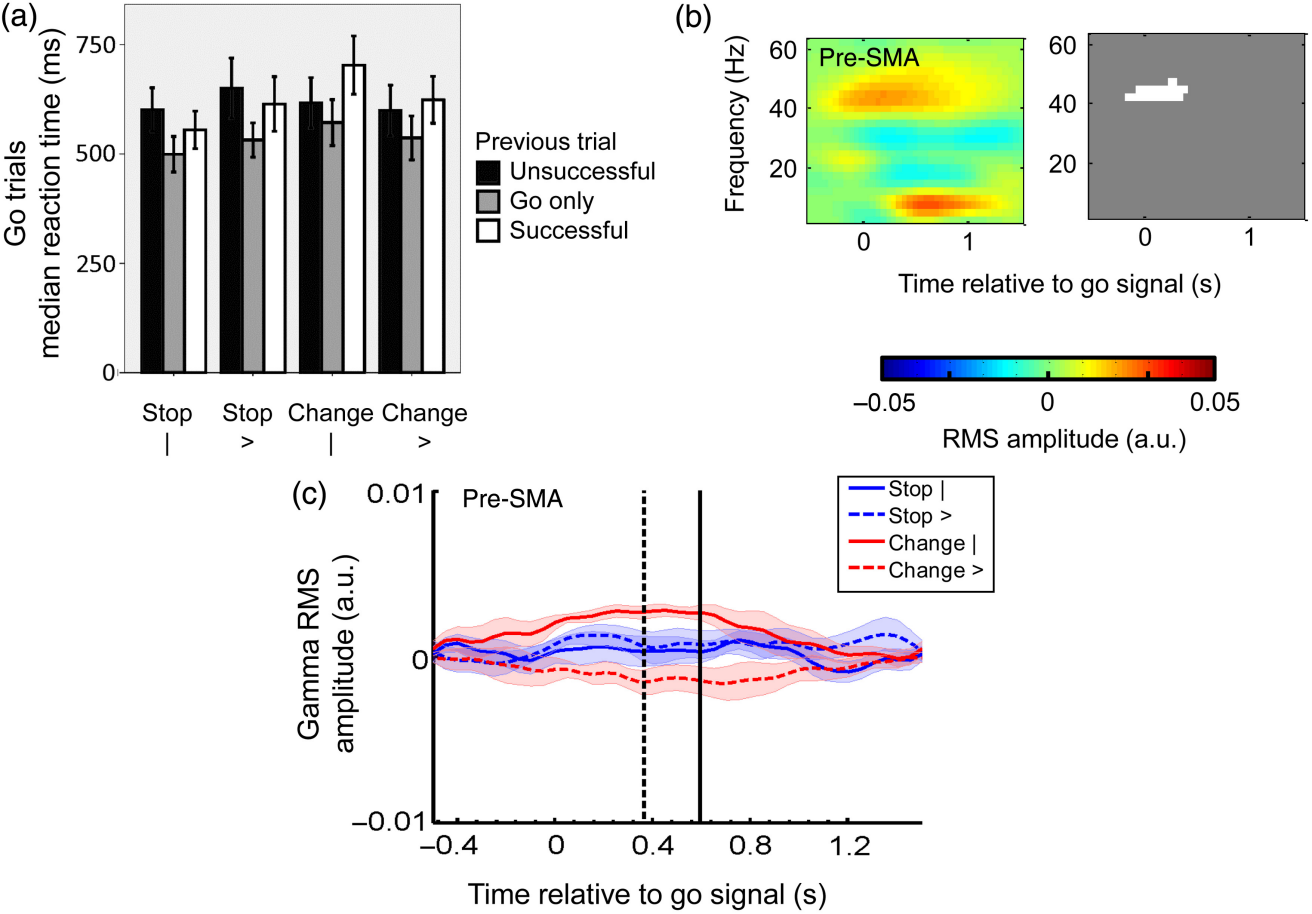
Contextual complexity and pre-SMA gamma activity. (*a*) Median Go only RTs are presented as a function of previous trial, response type, and Stop/Change-signal stimuli. Although the instructions for the Go task remain the same across and within task variants, 2 behavioral features are apparent. First, Go trials following Stop/Change trails are longer than average (main effect of previous trial (*F*_2,52.81_ = 12.50, *P* < 0.001). This effect is further explored in Figure 7. Secondly, more contextually complex task variants (Table 1) are more difficult and therefore have a longer Go task RT [a significant interaction (*F*_2,53.44_ = 4.27, *P* = 0.044) between Stop/Change cue and response]. (*b*) Time-frequency image showing the effect of complexity on the induced response to the Go signal in the pre-SMA. The grayscale image is a mask identifying significant increases (white) in RMS amplitude. More complex conditions result in increased gamma activity at the time of the Go signal (*t* = 0 s). The color scale represents the RMS amplitude in arbitary units. (*c*) The corresponding raw gamma activity averaged over subjects and conditions (from the convolution model). Behavioral markers showing timing of the median SOA (dashed black line) and the median RT of Go only trials (solid black line) have been overlaid onto the plot. Error bars represent ± standard error, including in (*c*) where standard error is given by the lighter shaded areas.

Modulations of contextual complexity were evident in behavior as increased Go RT, even after accounting for differential effects of previous trial type on current trial RT, such as the slowing in trials immediately following Stop-signals. The median Go RT was subjected to a mixed hierarchical GLM with Stop/Change stimulus (< or |), response (Stop or Change), and previous trial history (previous Go trial, previous successful Stop/Change trial, and previous unsuccessful Stop/Change trial) as factors. Here, because the contextual complexity of the Stop/Change cue is *reversed* for Stop versus Change trials, the effect of complexity is seen as a significant interaction (*F*_2,53.44_ = 4.27, *P* = 0.044; Fig. 4*a*) between Stop/Change cue and response. This result is particularly important because it confirms that subjects behaved according to our predicted rules (Table 1) where the major variations between tasks can be indexed by variations in conditional complexity, rather than any other potential interpretations. For example, one intuitive, alternative interpretation may view the conditional relationship between the Go signal and Stop/Change-signal as simpler than we have described—once the Go signal is given, any second signal means Stop/Change and therefore the shape/color of it or the type of response is unimportant. In such a scenario, however, subjects would respond equivalently in all Stop/Change task variants and therefore there would be no differences in Go RT—this alternative explanation is thus inconsistent with the behavioral data. Similarly, using only Stop/Change-signal color is inconsistent with our data as it would similarly predict no difference between individual variants of the Stop and Change tasks. Finally, these data confirm that complexity is evident behaviorally on Go only trials *even when the Stop/Change-signal is not manifest*. This is consistent with the idea that the complexity represents the contextual rules of the task (by definition the same in all trials), not the instantiation itself (i.e., it is not only a Stop-signal phenomenon). The effect of previous trial history was also significant—see later. No other effects (including the main effects of Stop/Change stimulus or response) were significant.

We also looked for evidence of the effect of contextual complexity on the SSRT; our prediction being that all task variants had the same requirement for inhibition and therefore there would be no effect of task variant on SSRT. We used a Bayesian MCMC procedure to estimate the inhibition function, taking into account RT drift, and determined the SSRT from its midpoint (Kuss et al. 2005; Fründ et al. 2011). The SSRT for each session was then subjected to a mixed hierarchical GLM with Stop/Change stimulus (< or |) and response (Stop or Change) as factors. Contextual complexity, the statistical interaction between the response and stimulus, did not affect the SSRT (*F*_1,20_ = 0.30, *P* = 0.592), supporting our assumption that all tasks had similar requirements for inhibition. No other effects were significant. Therefore, we were able to behaviorally dissociate the modulations of contextual complexity from the Stop process itself (as indexed by SSRT).

To look for the electrophysiological correlate of the contextual complexity, we applied a similar model to MEG data. Cortical time-series data were extracted from a priori defined regions of interest using an LCMV beamformer (Van Veen et al. 1997). We examined not only the right IFG and pre-SMA, but also other regions involved in the stopping network (the SMA and primary motor cortices; Aron et al. 2007; Nachev et al. 2007), and the left IFG to determine whether effects were regionally specific and/or unilateral (Fig. 2*a*). Note that the maximal mean percentage of variance explained was low (Fig. 2*b*), confirming that the source reconstruction allowed us to discriminate between sources of interest. Induced RMS amplitude responses to the primary go stimulus were estimated by a convolution model and converted to time-frequency images in peristimulus time (from 0.5 s before to 1.5 s after each stimulus; Fig. 3). We subjected these time-frequency images to a within-subject ANOVA with previous trial type (successful Stop/Change, unsuccessful Stop/Change, and Go only), Stop/Change stimulus (< or |), and response (Stop or Change) as factors. Two-tailed tests of main effects and interactions were thresholded at *P* = 0.05 FWE. Pre-SMA activity has previously been attributed purely to variation in motor preparation times or time on task (Grinband et al. 2011), but by including drifts in RT in our convolution model, we effectively removed the linear component of these confounds prior to statistical analysis of time-frequency images. There was a significant effect of contextual complexity (the interaction between Stop/Change stimulus type and response) on the induced response to the primary go signal only in the pre-SMA (Fig. 4*b*,*c*). This effect was in the gamma band and began at the time of trial onset, in keeping with a “set” effect rather than being specific to Stop/Change trials. There were no significant effects of trial history on the Go-induced response.

### Stop/Change-Signals Are Associated with a Rapid, Global Theta/Alpha Synchonization

**Figure 5.**
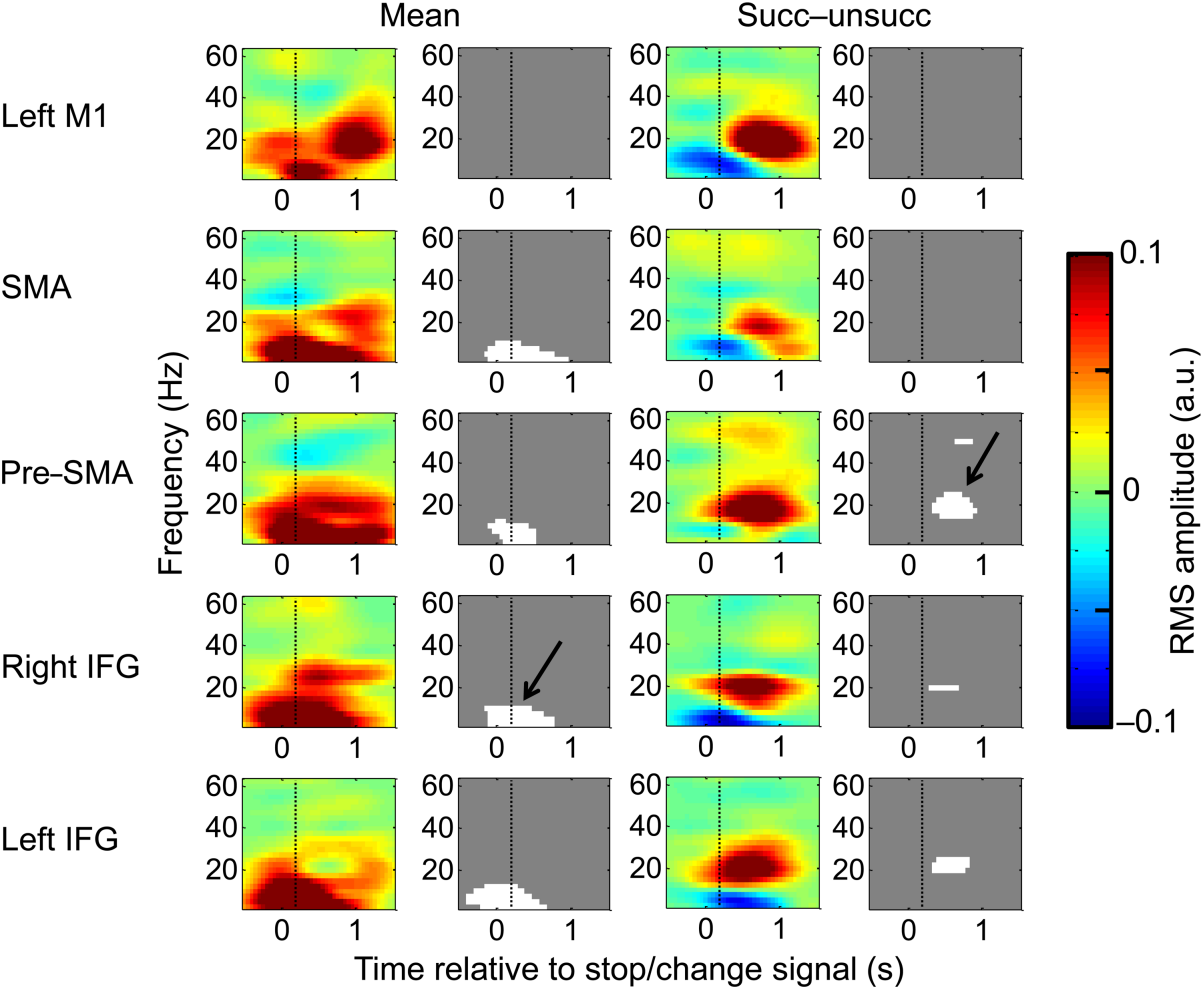
Time-frequency SPMs triggered to the Stop/Change-signal. Each subimage displays RMS amplitude changes associated with the Stop/Change-signal as a function of frequency (*y*-axis, Hz) and peristimulus time (*x*-axis, s, the Stop/Change stimuli occurs at *t* = 0). Each row contains information from separate cortical areas. Images are in pairs: the color image is the contrast image, while the grayscale image is a mask identifying significant increases (white) and decreases (black) in RMS amplitude triggered to the Stop/Change-signal. The first column displays the mean induced response to the Stop/Change-signal across all conditions (labeled “Mean”), with the associated statistical maps on the right. The third column displays the difference image between successful and unsuccessful Stop/Change trials (labeled “Succ − unsucc”) and the associated statistical maps on the right. Two major frequency patterns are visible (black arrows): A global theta increase around the time of Stop/Change-signal presentation, and a later beta increase in successful Stop/Change conditions restricted to more frontal regions. The color scale represents the RMS amplitude in arbitary units. M1: primary motor cortex; SMA: supplementary motor area; IFG: is inferior frontal gyrus.

**Figure 6.**
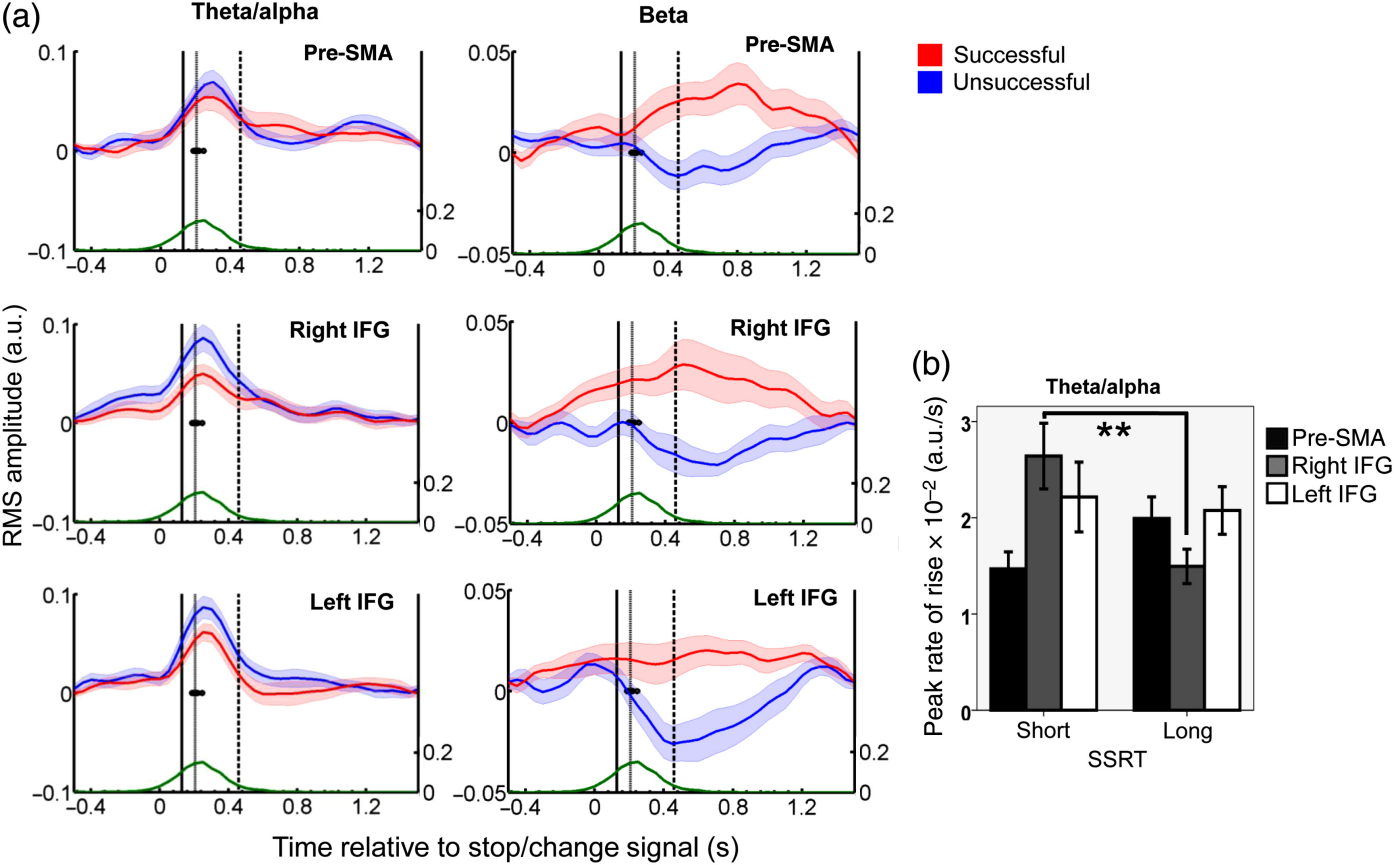
Timing of significant theta/alpha and beta RMS amplitude changes induced by the Stop/Change-signal. (*a*) The theta/alpha (2–12 Hz, left) and beta (15–25 Hz, right) raw RMS amplitude estimates (from the convolution model) have been averaged over subjects and conditions, and are represented as a mean (dark lines) and standard error (shaded area) activity over time (*x*-axis). Different rows display the activity of different cortical sources during successful (red) and unsuccessful (blue) Stop/Change trials. Behavioral data have been overlaid onto these plots: The median timing of button presses is presented for unsuccessful Stop/Change trials (a solid black line), and successful Change trials (dashed black line). The median SSRT is presented as a gray line, while the cumulative Go only RT distribution (after the mean SOA has been subtracted) is plotted as a green line with a separate *y*-axis (right). Units are arbitary units of RMS amplitude. (*b*) Peak rate of a theta/alpha rise in different cortical locations. The peak rate of rise of the theta/alpha response is steeper for efficient (short SSRT) when compared with less efficient stopping/changing (long SSRT) in the right IFG only (**: *F*_1,22_ = 10.334, P = 0.004). The peak rate of theta/alpha is presented as arbitary units of RMS power per second.

Because all Stop-signal task variants have the same requirement for stopping, we predicted that mean cortical responses associated with stopping would be similar across all variations in the Stop/Change task. Stop/Change-induced responses were estimated by the convolution model and converted to time-frequency images in peristimulus time (from 0.5 s before to 1.5 s after the Stop/Change stimulus). These time-frequency images were subjected to a within-subject ANOVA with success (successful or unsuccessful), Stop/Change stimulus (< or |), and response (Stop or Change) as factors. The mean response to a Stop/Change-signal was a global theta/alpha RMS amplitude increase that was significant in all areas except primary motor cortex (Fig. 5). For this theta/alpha response to be considered as a potential correlate of the Stop-signal, it must meet two requirements. First, it must occur *before* the SSRT, and secondly the signal must be *different* on unsuccessful as opposed to successful Stops/Change trails. This theta/alpha activity begins almost immediately after the Stop/Change-signal and certainly before the SSRT (Fig. 6*a*). When examining for the difference between successful and unsuccessful trials, no significant difference in amplitude was detected, but there was a temporal difference. On unsuccessful Stop/Change trials, the Go process was faster than average (see Table 2, as predicted by the horse-race model), and completed before the theta/alpha response reached its maximum. This can be seen in Fig. 6*a*—the theta/alpha response is just beginning when the average unsuccessful task button press occurs (marked by a solid black line). An interpretation would be that although the cortical signal to stop is generated, it arrives too late. We have previously shown that experimental modulations did not affect the behavioral measure of stopping—the SSRT. Similarly, the task stimuli, response requirements and conditional complexity did not significantly affect the theta/alpha component of the induced response (the electrophysiological correlate of stopping).

### Right IFG Theta/Alpha Activity Is Associated with the Length of the Stopping Process

These results suggest that a global theta/alpha response time-locked to the Stop/Change-signal may be causal to inhibiting an action. If this is correct then such activity should be related to behavior: theta/alpha activity should be more efficient (ramp more quickly) during sessions which have a shorter SSRT. We therefore divided subjects and conditions into those that had a shorter than average and those that had longer than average SSRTs. We calculated the peak rate of rise of the theta/alpha activity in a −0.2- to 0.5-s window relative to the Stop/Change-signal (value averaged across successful and unsuccessful Stop/Change trials), in the right and left IFG and the pre-SMA, and entered this into a mixed hierarchical linear model with source location (left IFG, right IFG, and pre-SMA), and average SSRT (long or short) as factors (Fig. 6*b*). Neither SSRT (*F*_1,66_ = 1.46, *P* = 0.232) nor source location (*F*_2,66_ = 1.47, *P* = 0.239) significantly affected the mean rate of theta/alpha rise. However, the interaction was significant (*F*_2,66_ = 5.32, *P* = 0.007). Post hoc analysis confirmed that the right IFG alone was significantly affected by SSRT (*F*_1,22_ = 10.334, *P* = 0.004). These results suggest that although the theta/alpha-induced response is global, the rate of rise of cortical activity in the right IFG is coupled most closely with the duration of the actual stopping process (defined as the SSRT). We performed further analysis to support the hypothesis that the theta/alpha response was compatible with a Stop/Change-signal and different to induced responses to other visual stimuli. We have added this as a Supplementary Result.

### Post Stop-Signal Slowing May Be Related to Frontal Beta Activity

**Figure 7.**
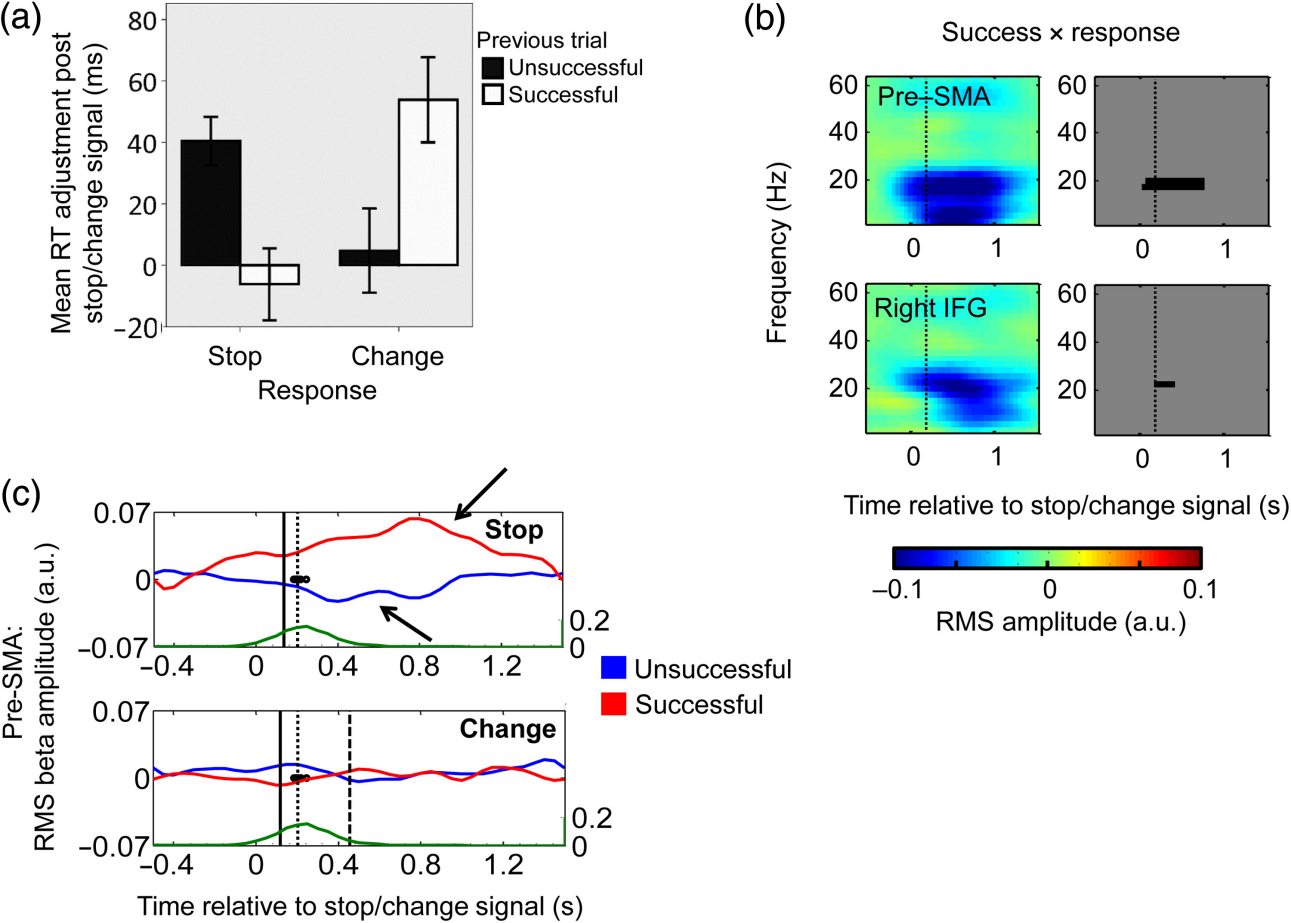
Post Stop/Change beta changes. (*a*) Post Stop/Change-signal Go only RTs adjusted for the previous Go only RT as a function of response type and success of the Stop/Change trial. There is greater post-stop slowing after an unsuccessful Stop trial, but greater post-Change slowing after a successful Change trial [interaction between response and previous trial (*F*_1,34.97_ = 17.10, *P* < 0.001)]. Error bars represent ±standard error. Time-frequency SPMs (*b*) and beta RMS amplitude plots (*c*) showing the electrophysiological correlate of the interaction between success and response in the pre-SMA and the right IFG. Figure conventions and behavioral markers are the same as for Figures 2 and 3. A significant beta power decrease can be seen in both areas following the Stop/Change event. RMS amplitude in pre-SMA over time is shown separately for Stop (top plot) and Change (bottom plot) conditions and for successful (red) and unsuccessful (blue) trials, to highlight the success × response interaction. Beta activity rises after successful stopping, drops after unsuccessful stopping (black arrows), but does not change during change trials. Activity in the right IFG (not shown) shows a similar pattern. Units are arbitrary units of RMS amplitude.

A further area of interest is the strategic changes that occur on the trial *after* a Stop/Change-signal trial. For example, does the subject become more cautious (slower) after seeing a Stop/Change-signal? Stop/Change-signals caused a significant post Stop/Change-signal trial slowing of Go RT [main effect of previous trial (*F*_2,52.81_ = 12.50, *P* < 0.001), Fig. 4*a*], and there was also a suggestion that the pattern of slowing after successful versus unsuccessful Stop trials was reversed for Change trials, although this effect did not quite reach significance [interaction between previous trial and response (*F*_2,52.81_ = 3.01, *P* = 0.058, Fig. 4*a*)]. However, post Stop-signal slowing estimates are confounded if different conditions lead to different amounts of RT drift (Bissett and Logan 2011). We addressed this by re-analyzing *only* post Stop/Change Go trials—we corrected their individual RTs by subtracting the RT of the preceding Go trial to remove drift [similar to Bissett and Logan (2011)]. After drift correction, the interaction between response and previous trial became significant (*F*_1,34.97_ = 17.10, *P* < 0.001; Fig. 7*a*). Subjects tended to slow their Go RT after an unsuccessful (as opposed to successful) Stop or a successful (as opposed to unsuccessful) Change.

There was a parallel effect on post Stop/Change-signal-related frontal beta activity. The effect of success (successful − unsuccessful) on the Stop/Change stimuli-induced response revealed increased beta-band activity in the pre-SMA, and right and left IFG (Figs 5 and 6*a*). This difference was most-marked in Stop trials, and hardly present in Change trials [interaction between success and the response was also significant in the same time-frequency region (Fig. 7*b*,*c*)]. However, the timing of this effect was probably too late to *influence* the majority of successful Stop/Changes—rather it is consistent with a *subsequent* error detection or network reorganization process. If so, the behavioral correlate of such neural activity should manifest on the *next* trial—that is, as a modulation of Go RT manifesting as post Stop/Change-signal slowing. Therefore, the post Stop-signal-induced beta response go part way in explaining the variations in post Stop-signal slowing that we found: A greater reduction in beta following an unsuccessful Stop, when compared with an unsuccessful Change, is associated with a greater increase in subsequent Go-task RT, whereas post successful Stop beta is *larger* than that of post successful Change beta, with a corresponding decrease in subsequent RT.

## Discussion

In this study, we set out to dissociate the functions of the pre-SMA and the right IFG while stopping a prepared movement. We designed 4 variants of the Stop-signal paradigm that allowed us to modulate the context of the task, while keeping the inhibited response the same (a button press). We found that neural activity underlying stopping was dissociated spatially, temporally, and spectrally into two components: increasing contextual complexity of the task (the complexity of the conditional rules governing when to Stop/Change) was associated with an early persistent gamma response in the pre-SMA, while the process of stopping itself (indexed by the SSRT) was closely related to the Stop/Change-signal theta-/alpha-induced response in the right IFG. Additionally, beta activity in the left and right IFG and pre-SMA partially reflected post Stop-signal behavioral changes.

We modulated the context, specifically the contextual complexity, of stopping in 2 ways: First by changing the response required from a Stop to a Change (which still includes stopping the Go task) and secondly by changing the Stop/Change stimuli cue from a vertical bar to a directional arrow. Crucially, the conditional link between the Stop/Change-signal and response required (a contextual feature we term conditional complexity defined as the minimal number of informative contextual elements that need to be combined in order to successfully stop or change) is reversed for stopping versus changing: A vertical bar results in a more complex Change, but a simpler Stop. Therefore, the interaction between the response required and Stop/Change cue *isolates* the effect of conditional complexity while controlling for confounds related to the Stop/Change stimulus and the type of response required. Behavioral measures were in keeping with this because more contextually complex tasks had significantly longer RTs (i.e., a significant response × signal interaction, see Fig. 4*a*). This effect was true of RTs even after taking into account post Stop/Change-signal effects on RT, suggesting that the effect of complexity is a set effect that applies to all trial types rather than a subset (e.g., post error trials only). Conditional complexity did not alter the measure of the duration of the Stop process—the SSRT.

The neural correlate of contextual complexity was identified as an early and persistent gamma response found in the pre-SMA. This feature is particularly interesting as gamma responses generally represent local cortical processing (e.g., Swettenham et al. 2009) rather than network processing, and early gamma activity in the pre-SMA has been seen in a conditional variant of the Stop-signal task in a single subject with subdural electrodes over the medial frontal wall (Swann et al. 2012). However, previous studies have conflicted as to whether neuronal firing in the pre-SMA differentiates successful and unsuccessful Stop/Change trials early enough to be potentially causal (Isoda and Hikosaka 2007; Scangos and Stuphorn 2010). Our findings are separate from this debate: pre-SMA gamma activity increased with increasing contextual complexity on *all* trials prior to the presentation of the first go signal and did not differentiate later success or failure at stopping. For similar reasons, it is unlikely that this effect can be explained by an increase in working memory. The size of the gamma response corresponding to the complexity effect is uniform across trials whether the subject is required to maintain a single piece of information (a go trial) or more (a Stop/Change trial), and the temporal profile of this response does not increase following the presentation of a second Stop/Change cue. Finally, recent fMRI studies have suggested that medial frontal activity is most parsimoniously related to “time on task” rather than experimental manipulations of conflict or error rate (Grinband et al. 2011; but see also Yeung et al. 2011). By using a convolution framework, we were able to estimate the effects of contextual complexity on pre-SMA activity *after* removing the linear effects of “time on task” and corresponding RT drift. Here, we assume that the non-linear effects are negligible in relation to the noise. We are not aware of any standard electrophysiological methods [but see Friston et al. (1998) for fMRI) that could address this problem in our framework. However, we have previously shown that as long as different events are processed by different populations of neurons, the true responses can be well recovered in the linear framework (Litvak et al. 2012). Therefore, our results are unlikely to be confounded by simple differences in RTs.

The most consistent response to the Stop/Change-signal was a brief theta-/alpha-induced response which peaked around the same time as the SSRT. This response was found in the pre-SMA, left and right IFG, and SMA (but was not significant in the primary motor cortex itself). It is unlikely that the spatially diffuse nature of this response is due to methodological confounds such as volume conduction or correlated lead fields, because the maximal mean squared correlation coefficient of the beamformer filters between sources was small (Fig. 2*b*). We therefore believe that the Stop/Change-signal causes parallel activation of multiple hubs of a stopping network. Similar medial frontal theta has been shown to be a marker of cognitive interference (Nigbur et al. 2011) and to predict error-monitoring (Cavanagh et al. 2009). But, in our experiment, the theta response was unaffected by the presence of an error (unsuccessful Stop or Change trials), irrespective of the Stop/Change stimulus cue. So how can a ubiquitous theta/alpha response be related to stopping? For such a claim two conditions must be met: the activity must start early enough before the SSRT lapses (the theoretical length of the stopping process), and the activity must differentiate successful from unsuccessful Stops/Changes. We suggest that *all* attended Stop/Change cues elicit a theta/alpha response in response to the Stop/Change-signal, which begins well before the SSRT (Fig. 6*a*)—and that Stop/Change failures are trials where this response has not had time to develop. This is demonstrated when brain activity is averaged in response to the Stop/Change-signal. Faster than average Go trials finish relatively early, before the theta/alpha response has had time to reach its maximum, and are therefore unsuccessfully countermanded. Slower than average go trials finish after this activity has peaked (the RT inferred to be slightly higher than the median Go RT) and are successfully countermanded. Explaining successful and unsuccessful stopping behavior in terms of a *temporal* relationship to the induced response negates the requirement to find changes in response *amplitude* causal to stopping. This is in keeping with fMRI studies which have consistently found a global network activation in response to a Stop-signal, but have found it difficult to find amplitude differences between successful and unsuccessful trials (Aron and Poldrack 2006; Aron et al. 2007; but see Li et al. 2006).

We also looked for the region which was most sensitive to the duration of the stopping process (as indexed by SSRT). At each frontal cortical region, we modeled the rate of rise of the induced theta/alpha response as a function of SSRT. We hypothesized that, in subjects and conditions with a longer Stop process and therefore longer SSRT, the cortical theta alpha response would be less steep. This relationship was only significant in the right IFG, suggesting that this part of the cortical stopping network is most closely related to the execution of the Stop process. This is consistent with human lesion (Aron et al. 2003) and fMRI data (Aron and Poldrack 2006), which have correlated right IFG damage and blood oxygenation with SSRT. Our results have also highlighted that right, as opposed to left, IFG activity is most closely related to stopping. It remains unclear why, but this asymmetry exists regardless of the hand being used to stop (Konishi et al. 1999), and whether a hand movement or verbal response is being stopped (Xue et al. 2008), supporting the idea that the right IFG operates a fundamental operation in stopping.

We found a novel behavioral dissociation between the RT changes following stopping and changing—greater increases in the Go task RT occurred after unsuccessful Stops (rather than successes) and Change successes (rather than unsuccessful Changes). Go task RT during the Stop-signal task has been reported to be lengthened after successfully inhibited trials (Emeric et al. 2007), after unsuccessful inhibited trials (Schachar et al. 2004), and after both (Rieger and Gauggel 1999; Bissett and Logan 2011), suggesting that undefined variations in the task or subject group on those studies may have led to different behaviors. In our study, the brain signals underlying these responses are less clear. Post Stop/Change-signal beta activity was significantly greater in successful than unsuccessful trials in the pre-SMA, and left and right IFG, but primarily *after* the SSRT. This response was significantly stronger for post Stop-signal responses and almost absent in the Change condition. This has two implications. First, the relative absence of beta changes in successful Change trials suggests that it is not a *necessary* cortical response for stopping. Secondly, beta activity may play a specific role when the subject requires only a Stop, and not a further response. So what role does cortical beta activity play in stopping? If it was purely an error signal, there would be no change in beta after a successful stop—this is not the case, and therefore we favor the idea that the beta increase after a successful Stop reflects reinforcement of the current motor program (Engel and Fries 2010; Jenkinson and Brown 2011), while the relative beta reduction following an unsuccessful response represents motor reprogramming (Tan et al. 2014). However, we did not find similar cortical responses to explain the behavior in the Change-of-plan paradigm, suggesting that post Change responses may be modulated by a different mechanism (e.g., locked to the motor response rather than the Change-signal).

In this study, we modulated the contextual complexity of stopping using 4 variants of the Stop-signal task. We found that the left and right IFG and pre-SMA were sensitive to the presence of a Stop-signal, and that a theta/alpha synchronization in these areas was early enough to be temporally causal to stopping. Of these areas, the right IFG was most closely associated with the duration of the stopping process as indexed by SSRT. Gamma activity in the pre-SMA was sensitive to modulations in contextual complexity. We additionally found post Stop-signal cortical responses that may explain strategic Go task RT changes in the Stop-signal task. Taken together, our findings define a distributed stopping circuit in which different features are preferentially expressed in specific temporally, spatially, and spectrally defined activities.

## Supplementary Material

Supplementary material can be found at: http://www.cercor.oxfordjournals.org/

## Funding

This study was funded by Parkinson's UK (F-0903 to A.J. and P.B.); the Wellcome Trust (to P.N.); the UCLH BRC (HICF-R9-501 to P.N.); the Medical Research Council (G0901503 to P.B.); Rosetrees Trust (to P.B.); the NIHR Biomedical Research Centre, Oxford (to P.B.), and the MRC/EPSRC UK MEG Partnership award (MR/K005464/1 to V.L. and G.B.); The Wellcome Trust Centre for Neuroimaging is supported by core funding from the Wellcome Trust (091593/Z/10/Z). Funding to pay the Open Access publication charges for this article was provided by the Wellcome Trust.

## Supplementary Material

Supplementary Data
