# Supplementary material for: The Frontal Control of Stopping
Source: Cereb Cortex. 2015 Mar 9;25(11):4392–406. doi: 10.1093/cercor/bhv027 (PMC4813761; doi:10.1093/cercor/bhv027)
Supplement: Supplementary Data [file supp_25_11_4392__index.html]

The Frontal Control of Stopping — The Frontal Control of Stopping — Supplementary Data 

# The Frontal Control of Stopping

## Supplementary Data

Supplementary Data

**Files in this Data Supplement:**

- Supplementary Data - Docx file
