# Supplementary material for: The Frontal Control of Stopping
Source: Cereb Cortex. 2015 Mar 9;25(11):4392–406. doi: 10.1093/cercor/bhv027 (PMC4813761; doi:10.1093/cercor/bhv027)

**Title: The frontal control of stopping - Supplementary material**

Ashwani Jha, Parashkev Nachev, Gareth Barnes, Masud Husain, Peter Brown, Vladimir Litvak

**Supplementary methods – behavioural analysis**

We compared the SSRT obtained from this method with two standard techniques (the average difference and integration methods) to ensure that our inhibition function-based analysis could reproduce the SSRTs captured by the former.

The first standard approach was the “average difference” method which assumes that the proportion of unsuccessful Stop/Change trials is 50%. In such a scenario, the mean SOA is subtracted from the mean go RT resulting in the SSRT (Verbruggen and Logan 2009; Nelson et al. 2010; Boehler et al. 2012). However in our case, in spite of the tracking procedure, this assumption is not correct, therefore we also used a second, “integration”, method (Verbruggen and Logan 2009; Nelson et al. 2010; Boehler et al. 2012). Here the mean SOA is subtracted from an *adjusted* go RT value, representing the go RT indexed by the average error fraction. For example in a session with an error fraction of 0.4 (40% of Stop/Change trials were unsuccessful), the 40^th^ percentile Go RT (of rank ordered Go RT trials) is taken as the adjusted Go RT. In this way the SSRT is adjusted for varying proportions of Stop/Change trials across sessions. In our report we present the average (avSSRT) of these two methods (table 2) in a similar fashion to (Nelson et al. 2010).

We then calculated an inhibition function, adjusting for the non-stationarity of reaction times, using an approach similar to Nelson et al (Nelson et al. 2010). In effect, they calculated a sliding “local” reaction time average of neighbouring trials and removed this fluctuation from the original reaction times. We emulated this by fitting a cubic spline to the go only reaction time data of each session. This conveniently modelled reaction time fluctuations and additionally provided a *predicted* Go RT (see later why this is required for electrophysiological analysis) for successful Stop/Change trials – an estimate of how the subject would have responded on Stop/Change trials if they had been Go-only trials, based on the reaction times of neighbouring trials (Nachev P, unpublished, http://ukpmc.ac.uk/theses/ETH/445162). To estimate the inhibition function, we only used data from Stop/Change trials: traditionally trials with similar SOAs are binned together and then the proportion of successful Stops/Changes at each SOA (y axis) is plotted against the SOA (x axis). Both Bayesian and non-parametric (e.g. bootstrapping) methods have been used to then fit a sigmoid function to the data (Nelson et al. 2010). We used procedures to transform the absolute values of the inhibition function onto a standardised axis, compensating for variations in Go task reaction time and allowing for the use of standardised Bayesian priors and therefore a more direct comparison (Logan and Cowan 1984; Band et al. 2003; Nelson et al. 2010). For each Stop/Change trial we subtracted the SOA from the predicted Go spline (an estimate of local Go task RT) to compensate for gross fluctuations in reaction time. We then subtracted the average SSRT (derived from previous standard procedures, avSSRT) from this value (i.e. the final transformation was predicted go RT – SOA – avSSRT) (Logan and Cowan 1984). The resulting transformation ensures that the midpoint of the plotted function (SSRT) lies around 0s for all subjects regardless of variations in their individual SSRTs and therefore the same absolute prior distribution (a Gaussian centred on 0s) can be used for all subjects without unfairly affecting subjects with different reaction times/ SSRTs. The ratio of successful: total Stop/Change trials was calculated as a function of this transformed time value in 100ms bins. We fitted a sigmoid inhibition function to these data using a Bayesian Markov Chain Monte Carlo (MCMC) procedure, which is superior to other methods such as bootstrapping (Kuss et al., 2005; Fründ et al., 2011). The function was defined by four parameters (with standard prior distributions): the upper limit of the function or “lapse rate” (prior~β (1, 10)), the 50% point of the function (prior~Ν (0,30)), the “slope” at 50% of the function (prior~γ (2,400))), and the lower limit or “guess rate” (prior~β (1,10)). Calculating the Bayesian posterior for such a model is *analytically* intractable, but it can be approximated by the MCMC procedure, which generates many samples of the posterior according to a particular algorithm (Kuss et al. 2005). We generated 2000 samples, discarding the first 1000 samples, to estimate an approximation to the posterior of the inhibition function. In this scenario the time at the mid-point of the inhibition function is equivalent to the difference between the avSSRT and the SSRT as calculated by the inhibition function. We assessed the goodness-of-fit of the model by calculating the deviance, which is a generalisation of the sum-of-squares metric that applies to binomial data (Fründ et al., 2011). Subjects with erratic behaviour not conforming to task predictions of a horse-race model would be identified by a high deviance (set arbitrarily).

**Supplementary results**

We performed further analysis to support the hypothesis that the theta/alpha response was compatible with a stop/change signal.

To support the hypothesis that the theta/alpha response to the stop/change signal was different from the induced response to other stimuli, we compared the maximum value (the peak) of this response to the Go response. The repetition suppression hypothesis suggests that the response to the second stimuli (the stop/change cue) should be weaker if both stimuli are equivalent.

We calculated the peak rise of the theta/alpha activity in a -0.2 s to 0.5s window relative to the Go signal (value averaged across sides), in the right and left IFG and the pre-SMA. Mean peak theta/alpha activity following Go signals was much lower than following the stop/change signal at all locations of interest (pre-SMA: Go 0.10 a.u., stop/change 0.69 a.u. Right IFG: Go 0.008, stop/change 0.079 a.u. Left IFG Go 0.007 a.u., stop/change 0.083 a.u.). This confirms that the stop/change response is a much larger response than that generated by other cues such as a go cue.

We also repeated the rise-time analysis on the Go theta/alpha induced responses to verify that the effect we found was localised to the stop/change signal. We calculated the peak rate of rise of the theta/alpha activity in a -0.2 s to 0.5s window relative to the Go signal (value averaged across sides), in the right and left IFG and the pre-SMA. We entered these data into a mixed hierarchical linear model with source location (left IFG, right IFG and pre-SMA), and average SSRT (long or short) as factors. Neither SSRT (F(1,66)=0.083, p=0.774 nor source location (F(2,66)=0.605, p=0.549), nor the interaction (F2,66)=0.518, p=0.598) was significant. This confirms that the rise-time analysis effects are confimed to the stop/change signal induced response.

**Caption to supplementary figure**

**Figure S1: Behavioural data examples.** The top image shows the timing of various trial events during 150 sequential trials in one subject during a Stop-signal task. Green circles represent the reaction time during go only trials. A green cubic spline has been fitted through these trials to highlight reaction time drift. Half of the trials had a Stop-signal, after a delay (SOA) represented by a red circle. Again a spline has been fitted to the SOA to visualise drift. Note how the two SOA staircases converge during the first few trials. Finally blue circles represent reaction times during unsuccessful Stop trials. Although there is some drift to both go reaction time and SOA, the resulting Stop/Change “decision time” (go only spline – SOA spline), a “local” approximation of SSRT is stationary. The plots beneath show the transformed inhibition functions derived by a Bayesian MCMC procedure for the same dataset (left) and a separate run (right). The plot on the left shows the proportion of successful responses to the Stop-signal as a function of predicted RT (the Go RT spline) – SOA – avSSRT. This transformation takes the original inhibition function (the proportion of correct Stop/Change responses on the y axis as a function of the SOA delay on the x axis), removes unwanted variance due to reaction time drift and centres the function on the SSRTav (the SSRT calculated using the traditional method). The data are represented by blue circles, with larger trials numbers resulting in larger circles. The mean fitted psychometric function is displayed by a blue line, with 20 alternative samples from the distribution of the function also presented (lighter blue corresponding to a lower likelihood). The midpoint of the function, equivalent to the difference between the SSRT calculated by this procedure (SSRTmcmc) and SSRTav, is highlighted with a red circle and is around 0s. The condition on the left shows a good fit, whereas the condition on the right shows a poor fit – the latter was rejected from further analysis (see table 2).


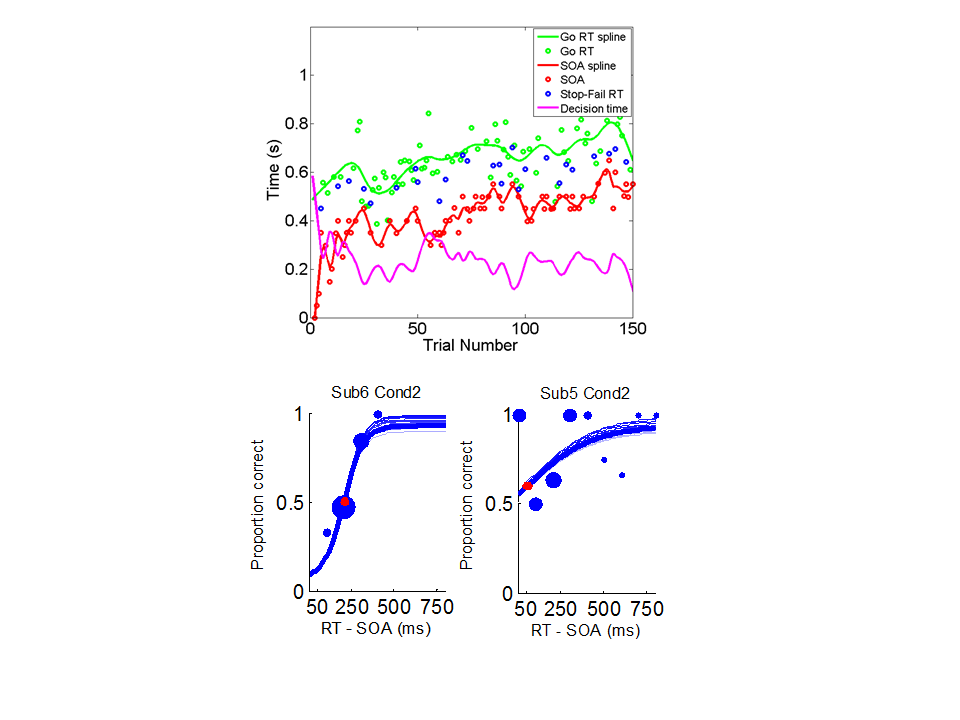

Supplement: Supplementary Data [file supp_bhv027_bhv027supp.docx]
